# Difficult Feeding Cases in Infants

**Published:** 1913-06

**Authors:** 


Difficult Feeding Cases in Infants. Chas. Edgerton Carter, M. D., Salt Lake City, *N. W. Med.*, April, 1913.

1. If malodorous, stools are usually indicative of: (a) Excess proteid. (b) Need for entire food revision.
2. If green with mucus (often sour smelling), usually indicative of: (a) Too high sugar. (b) Too high fat.
3. If normal color with curds and history of colic: (a) Too high proteids. (b) Excess fat. (c) Faulty methods.
4. Irritative stools: (a) Too high sugar. (b) Lack of lime salts. (c) Excess of fat.
5. Hard dry stools: (a) Too high proteid. (b) Wrong kind of proteid. (c) Too low fat.
6. Blood: (a) Irritative. (b) Pathognomonic.

A few general rules underlying all abnormal cases may be of service and fit into our feeding suggestions at this point.

1. Orange juice as a laxative rarely agrees before fifth month and, to avoid fermentation, should always be given between feedings.
2. Starch digesting enzyme rarely is active until fourth month.
3. Lime water constipates and, retarding peristaltic activity, may produce intestinal gas.
4. Abdominal chilling or habitually cold feet may produce colic and retard digestion.
5. Live enzymes of breast milk or fresh cow's milk are superior to "dead" or canned feeding.
6. Six hours in open air daily is essential for satisfactory oxidization of artificial feedings.
7. Salt in small but constant quantities is necessary in all artificial formulae.

Breast milk shows a negligible variation in the sugar content. Six to seven per cent. is constant, no matter what the mother's age, temperament, condition or period of lactation, while fat and proteid percentages vary markedly, particularly the fat. These three facts afford an invaluable key to the solution of difficult feeding cases, viz:

1. Let the sugar percentages approximate normal as the non-fluctuating factor in the food equation.
2. Let the proteid percentages vary in strength and form to meet conditions, but decrease and adjust the fat percentages with freer latitude.
3. Lactose, and maltose-dextrin never disagree when needed in the systemic economy. Proteid properly adapted in kind rarely is responsible for the blame so commonly ascribed to even low percentages, but fat with its propensity to ferment and cause colic needs careful supervision.

Czerny says "fat contained in milk is by far the most toxic ingredient." From that very conviction he elaborated the malt soup mixture. It is true that an increase in fat percentages raises the caloric total rapidly and for that reason appeals in certain marantic infants, but two practical deductions should be borne in mind:

1. That fat percentage increase in delicate children must be advanced with great caution up to normal, or the limit of tolerance.

2. We must practically double the amount of carbohydrates and proteids if we secure the same caloric values that a smaller quantity of fat contains.

To give the pith of such deductions in a sentence, young infants take high sugar values well, *e. g.*, malt, milk or cane sugar, while older infants require and will digest the higher proteids, such as gruel, malted, partially digested or plain.

